# Nintendo^®^ Wii Therapy Improves Upper Extremity Motor Function in Children with Cerebral Palsy: A Systematic Review with Meta-Analysis

**DOI:** 10.3390/ijerph191912343

**Published:** 2022-09-28

**Authors:** Desirée Montoro-Cárdenas, Irene Cortés-Pérez, María del Rocío Ibancos-Losada, Noelia Zagalaz-Anula, Esteban Obrero-Gaitán, María Catalina Osuna-Pérez

**Affiliations:** Department of Health Sciences, Faculty of Health Sciences, University of Jaén, Campus Las Lagunillas s/n, 23071 Jaén, Spain

**Keywords:** Nintendo^®^ Wii, videogames, cerebral palsy, upper extremity, grip strength, hand dexterity, arm functional movements, meta-analysis

## Abstract

Background: Nintendo^®^ Wii-based therapy (NWT) is a non-immersive virtual reality therapy used to recover upper extremity (UE) motor function in children with cerebral palsy (CP). We aimed primarily to elucidate the effectiveness of NWT in improving UE motor and functional impaired abilities in children with CP, compared to conventional therapy or no intervention. The secondary aim was to assess if NWT is more effective when used alone or combined with conventional therapy. Methods: A systematic review with meta-analysis was conducted from a bibliographic search in PubMed, Scopus, PEDro, Web of Science, and CINHAL, ending in October 2021, in accordance with PRISMA guidelines. We included randomized controlled trials that compared NWT vs. conventional therapy or no intervention in terms of their impact on different UE impaired abilities (grip strength, tip grip strength, UE dissociated movements, functional capacity in daily living activities, gross and fine motor dexterity, and grasping ability) in children with CP. Effect size was calculated with standardized mean difference (SMD) and its 95% confidence interval (95% CI). Results: Nine studies (276 participants) were included. NWT is more effective than conventional therapy at improving grip strength (SMD = 0.5, 95% CI 0.08, 0.91), tip grip strength (SMD = 0.95, 95% CI 0.3, 1.61), and grasping ability (SMD = 0.72, 95%CI 0.14, 1.3). NWT is more effective than conventional therapy at improving functional capacity in daily living activities (SMD = 0.83, 95% CI 0.07, 1.56). For fine manual dexterity, NWT was better than no intervention (SMD = 3.12, 95% CI 1.5, 4.7). Conclusions: Our results indicate that NWT is effective at improving various UE impaired motor skills in children with CP.

## 1. Introduction

Cerebral palsy (CP) is defined as a group encompassing permanent, changeable movement, posture, and motor function disorders [1,2] which arise as a consequence of damage to the developing fetal or infant brain [3]. Currently, CP is one of the major causes of childhood physical disability [4], with an estimated prevalence of 2–3 cases per 1000 [5,6]. CP produces general disorders in muscle tone, joint stiffness, muscle weakness, and muscle pain [7], and decreases motor control [8]. These changes influence physical function [9] and are more pronounced in upper-extremity-related functional capacity, and even more so in hand dexterity [10]. Approximately 83% of children with CP have upper extremity (UE) motor disabilities, and 36% have wrist and hand muscle contractures [11]. The changes in motor function of the UE lead to difficulties in performing daily living activities [12] and self-care, negatively impacting the quality of life of these patients, and increasing the burden on their caregivers. It also limits children’s participation in family, school, and leisure activities [13]. Improving UE motor function may facilitate their daily living activities, and also improve their quality of life due to an increased involvement in social and educational environments.

Conventional therapy aims to recover UE motor function in individuals with central nervous system diseases [14,15]. Conventional therapy interventions include passive and active techniques that can increase UE muscle strength, flexibility, and hand dexterity, with the aim of maximizing motor function and reducing sequelae [16]. Some patients report that conventional therapy protocols are repetitive, lengthy, and monotonous, which can reduce motivation and adherence to the therapy [17,18]. In recent years, virtual reality (VR) has been used as a therapeutic option in the neurorehabilitation of central nervous system diseases [19,20], especially to restore balance [21] and UE motor function in children with CP [22]. VR devices allow patients to be included in and to interact with simulated environments that they recognize as real [23]. The interaction with this virtual environment can differ according to the level of immersion and presence of these devices, and non-immersive, semi-immersive, and immersive VR devices are available. Among the different VR devices, non-immersive VR devices are the most promising for incorporation into therapeutic programs [24], and Nintendo^®^ Wii-based therapy (NWT) is one of the most widely used in children with CP. In this system, patients perform movements and interact with virtual objects displayed on a screen using a remote joystick, with or without a platform called the Wii Balance Board™ [25]. In addition to other non-immersive VR systems, such as Sony PlayStation^®^, Xbox^®^ or Kinect^®^, NWT requires the active participation of the participant in performing different sports and recreational activities within the games in a standing up or sitting position and can adapt to the needs of each patient. Previous studies reported that NWT is easy to use, accessible, provides a way for children to socialize with their peers; and increases their motivation and leisure during therapy when it is added to conventional therapy [26,27]. Meldrum, D et al. (2012) found that approximately 88% of patients would include Nintendo Wii in their future treatments [28]. Furthermore, the use of NWT did not seem to produce side effects, unlike the dizziness, nausea, headache, etc., that seems could appear in immersive VR [29,30]. In addition, NWT is a low-cost, commercialized, and useful tool that allows the training of flexibility, balance, strength, and coordination in safe environments, such as homes or clinical centers [31,32,33]. 

To date, several systematic reviews have been carried out to analyze the effect of VR interventions (including non-immersive VR devices) on UE motor function in children with CP, with unclear conclusions due to the heterogeneity between studies in relation to the great variety of VR devices used [34,35,36]. Another handicap of the published reviews is the lack of a specific grouping of all the measurement instruments used, with the aim of identifying specific domains of UE motor function. We found that, in those reviews, NWT was the most widely used type of VR intervention, and extensive scientific reports have been written about it. Previous reviews assessed the effect of NWT in improving balance in children with CP [25,37], and in other neurological diseases, such as Parkinson’s disease [38,39] or stroke [29,40]. Thus, in comparison with other reviews that assessed all non-immersive VR devices, our aim was to assess whether NWT, as one of the most non-immersive VR devices available, had an effect on improving UE motor function abilities in children with CP. For this reason, we designed this systematic review with the following research question in mind: Is NWT more effective than conventional therapy or non-intervention when it comes to improving different UE motor function abilities in children with CP? First, we systematically searched all available databases to analyze the effectiveness of NWT in improving UE motor and functional impairments in children with CP, by checking its effect on different UE dimensions: grip strength and tip grip strength, functional capacity in daily living activities and self-care, UE dissociated movements, gross manual dexterity, fine manual dexterity, and grasping ability. Second, we specifically assessed whether NWT used as the only therapy or combined with conventional therapy was better than conventional therapy or no intervention at improving UE motor function in children with CP.

## 2. Materials and Methods

### 2.1. Report and Protocol Design

A systematic review with meta-analysis was conducted according to the Preferred Reporting Items for Systematic Reviews and Meta-Analyses (PRISMA) [41] and the Cochrane Handbook of Systematic Reviews of Interventions [42]. This review was previously registered in PROSPERO (CRD42021254778). 

### 2.2. Bibliographical Search

Two authors (D.M.-C. and E.O.-G.) independently performed a literature search in PubMed Medline, Scopus, Web of Science, PEDro (Physiotherapy Evidence Database), and CINHAL, finishing in October 2021. The authors examined the reference lists from the retrieved full-text studies, previously published reviews, and grey literature (congress abstracts and expert documents, among others). The PICOS tool proposed by the Cochrane Library [42] was used to identify potential studies based on population (children with CP), intervention (NWT), comparison (conventional therapy and/or no intervention), outcomes (UE motor function), and study design (randomized controlled trials [RCTs]). According to Medical Subject Headings (MeSH) and CINHAL Subjects Headings, the keywords employed in our search were “Nintendo^®^ Wii”, “virtual reality”, “cerebral palsy”, “upper extremity”, “hand”, “wrist”, “shoulder”, “arm”, and “elbow”, including other related MeSH and entry terms. For each database, a specific keyword combination was employed using the appropriate tags and the Boolean operators “and”/“or”. Filters related to publication date and language were not used. Concerns regarding the search strategy were raised with a third expertise author (M.C.O.-P.). Table 1 shows the search strategy used in each database.

### 2.3. Study Selection: Inclusion and Exclusion Criteria

The study selection process was carried out independently by two authors (D.M.-C. and E.O.-G.), who reviewed all retrieved records by title and abstract. Each study was examined in detail by two reviewers. All disagreements that arose during this phase were resolved by a third author (M.C.O.-P.). A study was only included in our review if it met all the inclusion criteria: (1) RCTs; (2) including subjects diagnosed with CP; (3) with at least two groups of intervention, in which one received NWT in comparison with conventional therapy or no intervention; (4) studies that assessed the different outcomes related to UE motor function; and (6) studies that provided quantitative data of the outcomes of interest to carry out the meta-analysis. The exclusion criteria established were: (1) RCTs with groups of patients with different central nervous system diseases (for example, heterogeneous groups of neurological patients that included children with CP); (2) outcomes that could not be unified when performing the meta-analysis; (3) RCT studies that did not provide sufficient data to be included in the quantitative synthesis of this review. 

### 2.4. Data Extraction

Two authors (D.M.-C. and I.C.-P.) independently extracted data from the studies included using a Microsoft Excel standardized data-collection form designed specifically for this research, similar to that used in previous meta-analyses [25,43,44]. Disagreements were resolved by the third author (M.C.O.-P.). 

Specifically, in this review, from each selected study, the characteristics collected were the following: (1) overall characteristics (authorship and publication date, study design, country); (2) participant characteristics (total sample size, number of groups, participants per group, age, sex ratio, type of CP, level of the Gross Motor Function Classification System [GMFCS], and time since diagnosis); (3) characteristics of the experimental and control intervention (type, number of sessions, number of weeks or months, number of sessions per week, and duration in minutes of each session); (4) data from outcomes of interest (mean and standard deviation); and (5) follow-up period (short-term, medium, or long-term follow-up). When a study did not provide standard deviations, it was estimated using standardized transformations through the standard error, range, interquartile range, and median [42]. 

### 2.5. Outcome Measures

The outcomes assessed in this systematic review with meta-analysis were the following: grip strength, tip grip strength, functional capacity in daily living activities and self-care, UE dissociated movements, gross manual dexterity, fine manual dexterity, and grasping ability. Table 2 summarizes information related to the outcome measures.

### 2.6. Risk of Bias and Quality of Evidence Assessment

The risk of bias in the studies selected was assessed through the Cochrane Collaboration Risk of Bias Tool. This scale comprises seven items (selection, performance, detection, attrition, reporting, and other bias), and gives the labels of low, uncertain, and high risk of bias [45].

The quality of evidence in each meta-analysis was evaluated using the Grading of Recommendations Assessment, Development and Evaluation (GRADE) system [46]. We followed the GRADE checklist proposed by Meader [47] to estimate the quality of evidence, taking into account the risk of bias of each selected study, inconsistency, inaccuracy, lack of directiveness, and risk of publication bias. The quality of evidence could be high (if our findings were robust); moderate (if our results changed, including new studies); low (if the level of confidence in our pooled effect was very slight); and very low (when some items were not present). The quality of evidence of each meta-analysis was downgraded from high quality by one level for each factor that we found. In the case of the presence of several limitations, the overall quality level was downgraded by two levels. Risk of bias of the individual studies and the quality of evidence assessments were carried out by two independent authors (M.d.R.I.-L. and N.Z.-A.), and disagreements resolved by a third author (E.O.-G.). 

### 2.7. Statistical Analysis and Additional Analyses

Comprehensive Meta-Analysis 3.0 (Biostat, Englewood, NJ, USA) [48] was used to perform the meta-analysis by two authors (E.O.-G. and I.C.-P.). The pooled effect was calculated using the Cohen’s Standardized Mean Difference (SMD) [49] that may be interpreted at three effect strength levels: small (SMD = 0.2), moderate (SMD = 0.5), and large (SMD ≥ 0.8) [50].When an outcome was assessed with data from studies that use the same measurement test, we calculate the mean difference (MD) to compare with the minimally clinically important difference (MCID) for that test. Forest plots were used to display our findings [51]. Risk of publication bias was analyzed visualizing the funnel plot (asymmetric indicates risk of publication bias, and symmetric low risk) [52] and with the *p*-value for the Egger test (where if *p* < 0.1 it indicates that risk of publication bias exists) [53]. In addition, we calculated the adjusted effect considering any possible publication bias, using the Trim-and-fill method [54]. To estimate the risk of publication bias, our software requires almost three studies, so sometimes could not be estimated. Finally, the inconsistency was assessed with the level of heterogeneity through the degree of inconsistency (I^2^) from Higgins (small <25%, moderate 25–50% and large ≥50%) and *p*-value for Q-test (*p* < 0.1 indicates presence of heterogeneity) [55,56].

## 3. Results

### 3.1. Study Selection

**Figure 1:**
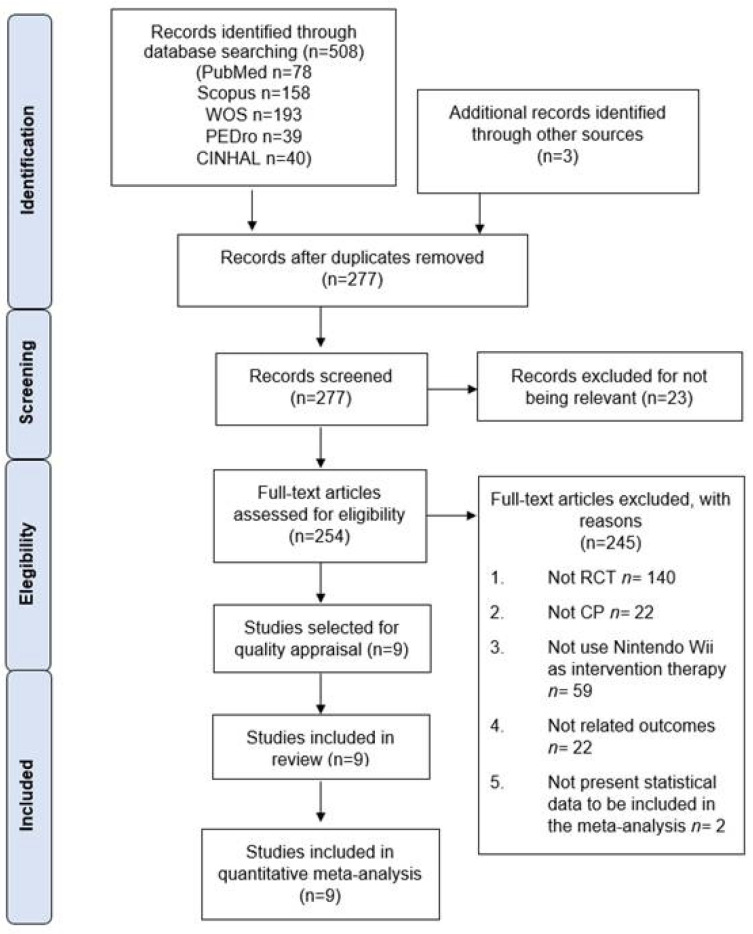
PRISMA flow chart of the study selection process.

Initially, 508 references were retrieved from databases after the bibliographic search, and a further 3 studies from other resources. After duplicate studies were removed, 277 studies were screened by title/abstract. Of these, 23 records were excluded for not being relevant and 245 references were removed for not meeting the inclusion criteria. Finally, 9 studies were included in the quantitative synthesis [57,58,59,60,61,62,63,64,65]. The PRISMA flow chart (Figure 1) summarizes the screening and study selection phase. 

### 3.2. Characteristics of the Studies Included in the Review

The studies included were carried out between 2012 and 2021 in Turkey [57,59,60], Saudi Arabia [58,62], Australia [61], India [63], South Korea [64], and China [65]. The studies included reported data from 274 participants (mean age 9.56 ± 2.35 years old and 48% girls). Eight studies [57,58,59,61,62,63,64,65] reported data from participants diagnosed with spastic, diplegic, or tetraplegic CP, and one study [60] reported non-specific CP. Related to GMFCS levels, 2 studies [57,59] included patients in levels I and II; one study [58] included patients in level III; 2 studies [60,63] included patients in levels I–IV; another study [61] included patients in all levels I–V; and 3 studies [62,64,65] did not report the GMFCS level. The experimental group consisted of 138 individuals receiving NWT or NWT + conventional therapy, and the control group included 136 individuals (conventional therapy or no intervention). Seven studies [57,59,60,61,62,63,65] compared the effect of NWT plus conventional therapy versus conventional therapy; one study [64] compared NWT versus conventional therapy, and another study [58] compared NWT vs. no intervention. The duration of the therapy in weeks was 6 weeks or less in two studies [57,63] and more than 6 weeks in seven studies [58,59,60,61,62,64,65]. The duration of each session was less than 30 minutes in three studies [57,58,59] and more than 30 minutes in six studies [60,61,62,63,64,65]. Four studies [57,59,64,65] applied the therapy twice a week; 3 studies [60,61,62] applied it three times a week; and 2 studies [58,63] applied it more than three times a week. Table 3 shows the main characteristics of the studies included in this review. 

### 3.3. Risk of Bias Assessment

Table 4 shows the risk of bias in the studies included. A high risk of selection bias was found in five studies [57,58,60,61,64]. No studies were able to blind the type of therapy to the CP participants, increasing the risk of selection bias. Five studies [57,58,59,60,65] did not blind the assessors after the treatment, increasing the risk of detection bias. In general, the overall quality of the included studies was moderate due to the possible presence of selection, performance, and detection bias.

### 3.4. Findings in Meta-Analyses

#### 3.4.1. Grip Strength

**Figure 2:**
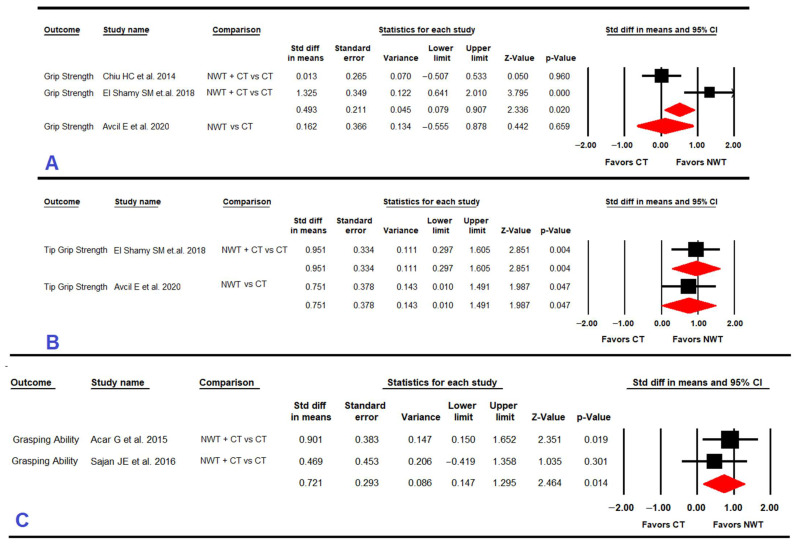
Forest plot effect of Nintendo^®^ Wii therapy on grip strength (**A**), on tip grip strength (**B**), and on grasping ability (**C**).

Three RCTs [60,61,62] reported data for 127 children with CP to assess the effect of NWT on grip strength (Table 5 and Figure 2). Our findings showed very low-quality evidence of a medium effect (SMD = 0.5; 95% CI 0.08, 0.91; *p* = 0.02) favoring NWT + conventional therapy vs. conventional therapy, with an increase of 1.8 points (95% CI 0.95, 2.63; *p* < 0.001) in grip strength according to dynamometer measurement. The heterogeneity level was moderate (I^2^ 52%; Q 8.9, df = 1; *p* = 0.003) and the precision level was also low (48.5 participants per study). When NWT was compared to conventional therapy, no difference was found between therapies for improving grip strength (SMD = 0.16; 95% CI −0.55, 0.88; *p* = 0.66). 

#### 3.4.2. Tip Grip Strength

Two RCTs [60,62] provided data for 70 children with CP to analyze the effect of NWT on tip grip strength (Table 5 and Figure 2). On the one hand, very low-quality evidence with a large effect (SMD = 0.95; 95% CI 0.3, 1.61; *p* = 0.004) favored NWT + conventional therapy vs. conventional therapy, without heterogeneity. On the other hand, very low-quality evidence with a large effect (SMD = 0.8; 95% CI 0.01, 1.49; *p* = 0.047) favored NWT vs. conventional therapy without heterogeneity.

#### 3.4.3. Grasping Ability

Two RCTs [57,63] reported data for 48 children with CP to assess the effect of NWT on grasping ability (Table 5 and Figure 2). The results showed very-low quality evidence of a medium-large effect (SMD = 0.72; 95% CI 0.14, 1.30; *p* = 0.014) favoring NWT+ conventional therapy vs. conventional therapy, with an increase of 4.35% (95% CI 0.96, 7.75; *p* = 0.003) in the “grasp” domain of the QUEST questionnaire. Heterogeneity was not found, and the precision level was low (24 participants per study).

#### 3.4.4. Functional Capacity in Daily Living Activities and Self-Care

**Figure 3:**
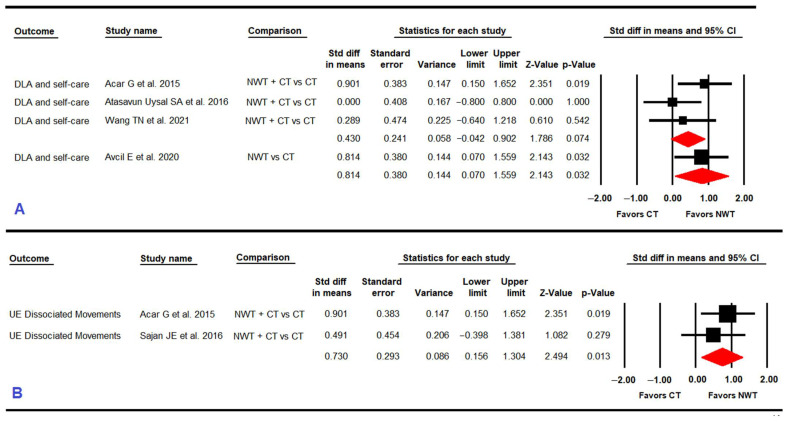
Forest plot effect of Nintendo^®^ Wii therapy on daily living activities (DLA) and self-care (**A**) and on upper extremity (UE) dissociated movements (**B**).

Four RCTs [57,59,60,65] reported data for 102 children with CP to assess the effect of NWT on functional capacity in daily living activities and self-care (Table 5 and Figure 3). No statistically significant differences (SMD = 0.43; 95% CI −0.04, 0.91; *p* = 0.074)) were found between NWT+ conventional therapy vs. conventional therapy without risk of publication bias (Egger *p* = 0.66 and 0% variation in the trim-and-fill method). However, very low-quality evidence of a large effect favored NWT (SMD = 0.82; 95% CI 0.07, 1.56; *p* = 0.032) in comparison with conventional therapy.

#### 3.4.5. Upper Extremity Dissociated Movements

Two RCTs [57,63] reported data from 48 children with CP to analyze the effect of NWT on UE dissociated movements (Table 5 and Figure 3). Our results showed very low-quality evidence of a medium-large effect favoring NWT + conventional therapy (SMD = 0.73; 95% CI 0.15, 1.3; *p* = 0.013) compared to conventional therapy, increasing the score of the domain “dissociated movements” of the QUEST questionnaire by 4.094% (95% CI 0.75, 7.43; *p* = 0.016). Heterogeneity was not found (I^2^ 0%; *p* = 0.33) and the precision level was low (24 participants per study).

#### 3.4.6. Gross Manual Dexterity

**Figure 4:**
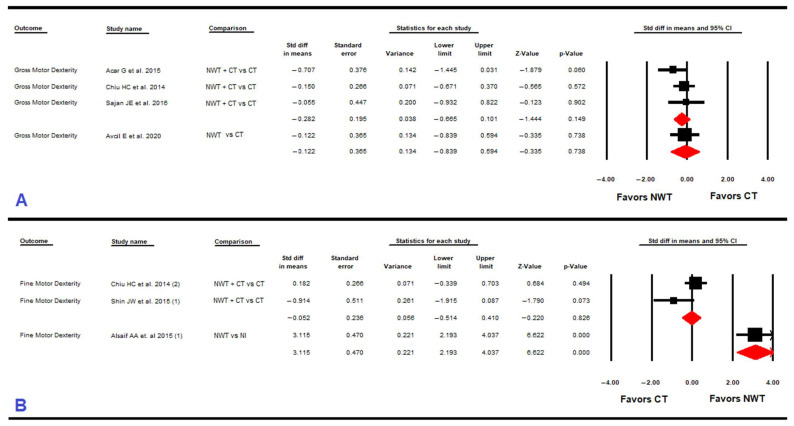
Forest plot effect of Nintendo^®^ Wii therapy on gross motor dexterity (**A**) and on fine motor dexterity (**B**).

Four RCTs [57,60,61,63] provided data for 135 children with CP to assess the effect of NWT on gross manual dexterity (Table 5 and Figure 4). No statistically significant differences between NWT+ conventional therapy vs. conventional therapy (SMD = −0.28; 95% CI −0.67,0.1; *p* = 0.149) and between NWT vs. conventional therapy (SMD = −0.12; 95% CI −0.84 to 0.6; *p* = 0.74) were found without risk of publication bias. Heterogeneity was not present in any subgroup, and the precision level in both comparisons was low (30 and 35 participants per study, respectively).

#### 3.4.7. Fine Manual Dexterity

Three studies [58,61,64] provided data for 114 children with CP to assess the effect of NWT on fine manual dexterity (Table 5 and Figure 4). No statistically significant differences (SMD = −0.05; 95% CI −0.51, 0.41; *p* = 0.82) were found between NWT+ conventional therapy vs. conventional therapy without heterogeneity. However, very low-quality evidence of a large effect (SMD = 3.12; 95% CI 1.53, 4.7; *p* < 0.001) favored NWT vs. no intervention without heterogeneity. The precision level in both comparisons was low (40 and 34 participants per study, respectively).

## 4. Discussion

The main aim of our meta-analysis was to evaluate the effect of NWT on UE motor function recovery in children with CP. We included nine RCTs that reported quantitative data from 274 subjects with CP to analyze seven abilities dependent on UE motor function. Our meta-analysis found the following: (1) NWT can be considered effective for the treatment of UE function in children with CP; (2) NWT plus conventional therapy is better than conventional therapy alone for recovering grip strength, tip grip strength, grasping ability, and UE-dissociated movements in children with CP; (3) NWT is better than conventional therapy to improve tip grip strength and functional capacity in daily living activities and self-care in children with CP; and (4) NWT is better than no intervention to improve fine manual dexterity in children with CP. Our study differs from previous reviews [34,66] as it is the first meta-analysis to assess the effect of NWT in this variety of UE motor dimensions. It is important to point out that, although this is a novel study, these results should be considered with caution due to the low number of studies included in each meta-analysis, which reduces the robustness of the results. However, taking into account the low level of evidence, this meta-analysis is the first that provide information about the use of NWT to restore UE motor-function in children with CP. Previous reviews assessed the effect of some non-immersive VR devices, such as NWT or custom video games, but did not specifically assess the effect of NWT protocols for these seven UE abilities. Previous studies such as that of Alrashidi M et al. analyzed the effect of VR on UE function in children with CP but, in contrast with our meta-analysis, they did not analyze the effect of a particular VR device, they included fewer trials and they only assessed two domains of UE function. Other reviews only reported the effect of all VR devices on global UE function, but we showed that it is necessary to assess the effect of this therapy for each specific domain of UE in children with CP. We consider that grip strength and fine manual dexterity are different skills, and the effect of NWT was likely to be different for each. NWT may be of interest to physical clinicians as it is cheaper, more accessible, and easier to use than other non-immersive VR devices, and it is important to analyze the effect that NWT can produce for different UE motor skills.

For grip strength, our results showed that NWT is effective when used as complementary therapy with conventional therapy, leading to an increase of 1.8 points in dynamometer measurement. For this variable, both therapies are effective separately, without significant differences between them. For tip grip strength, our results showed that NWT is effective when it is used as a single therapy and when it is used combined with conventional therapy, both in comparison with conventional therapy. The improvement in grip strength and tip grip strength may be due to grasping the Nintendo joystick and pressing its buttons to play and interact with the virtual environment [67]. The effect of NWT on grip strength was not assessed in previous reviews. As such, our results cannot be directly compared with other reviews as they did not specifically assess the effect of NWT on these outcomes. Only Alrashidi, M and colleagues included three RCTs (two of NWT) to assess the effect of non-immersive VR in improving grip strength in children with CP. However, although it appears that non-immersive VR is effective in improving grip strength, their review did not present meta-analyses and some important studies were not included, so their findings may be questionable, meaning that our findings are difficult to compare with them. In addition, in other previous meta-analyses that assessed the effect of the Leap Motion Controller, as an example of non-immersive VR, on grip strength [68], non-immersive VR was found to be useful to improve this variable in different neurological diseases, such as CP. Therefore, our results reinforce the use of non-immersive VR devices to increase grip strength in children with CP.

For grasping ability, our results showed that the use of NWT more conventional therapy is better than conventional therapy, with a medium-high effect. In this case, it was not possible to assess if NWT alone is better than conventional therapy or no intervention due to a lack of studies. The improvement in grasping ability can be also justified by the use of the remote control, as in grip strength and tip grip strength, since in order to perform the different functions requested by the games, it is necessary to hold the Nintendo^®^ Wii joystick constantly in a deliberate position between the hands. This continuous grip of the joystick and increase in grip strength may explain the improvement in grasping ability. This is of significant clinical interest as NWT may help patients with CP to better grip objects used in everyday life and may in this way increase personal autonomy and self-care.

The results obtained for functional capacity in daily living activities and self-care showed an improvement with a large effect for NWT compared to conventional therapy. When NWT plus conventional therapy was compared to conventional therapy, no significant differences were found between therapies. Both comparisons are at the limit of significance. Future studies will indicate if both therapies could be complementary. For now, the results indicate that NWT can be considered a good therapeutic option to improve functional capacity in activities of daily living and self-care. According to Avcil E et al. (2020), the improvements in this variable may be because the exercises performed in the chosen video games were similar to the gestures performed in day-to-day activities. The use of these video games, despite the fact that they were not developed for rehabilitation purposes, could indirectly produce an improvement in the gestures required for daily living activities. This could justify the use of NWT alone as a treatment option. 

For UE-dissociated movements, our results showed an improvement with a medium-large effect of the NWT more conventional therapy vs. conventional therapy. However, we could not analyze the effect of NWT vs. conventional therapy due to a lack of studies. Alrashidi, M and colleagues assessed the effect of non-immersive VR (NWT and Xbox) in UE abilities using QUEST, showing improvements in this outcome. However, it is difficult to compare our review with theirs, as our meta-analysis clarifies its findings, indicating that NWT combined with more conventional therapy is able to improve children’s UE abilities. Nintendo^®^ Wii games require widely differing movements related to the affected UE to correctly complete the virtual activities, and to complete the virtual tasks it is necessary to be able to dissociate both UEs. These movements are carried out with a joystick and displayed on a screen. The continuous and repetitive exposition and training of the UE movements in these virtual environments may explain the improvement in the ability to dissociate UE movements in children with CP.

When fine manual dexterity was assessed after application of NWT, our results showed that NWT is better than no intervention. However, we did not find a greater effect for NWT more conventional therapy or NWT over conventional therapy for fine or gross manual dexterity. Both types of therapies improve gross manual dexterity and fine manual dexterity, but neither therapy is superior to the other. Our results, although we only assessed NWT, may be in consonance with previous reviews that determined that non-immersive VR devices could be useful to improve fine manual dexterity in various non-traumatic neurological diseases, including CP [36,68]. 

The improvements that NWT can produce in the UE motor skills of children with CP may be based on neuroplasticity mechanisms and motor learning induced by multisensory stimulation during the use of NWT. Nintendo^®^ Wii games help to immerse patients in virtual environments and allow them to dynamically interact with situations and activities that are impossible in the real world. In neuroimaging studies carried out in children with CP, it has been observed that the use of VR induces neuroplasticity and causes changes at the level of the cerebral cortex [69]. Using NWT, activity-dependent plasticity is mainly achieved based on the execution of specific activities repeatedly and within certain intensity and time parameters, as well as multisensory stimulation [70,71]. It also produces improvements at the motor level with respect to conventional therapy, thus favoring the use of systems that relate to perception, memory, and learning in children with CP [72,73]. Various Nintendo^®^ Wii games could comply with the principles of neuroplasticity and motor learning indicated above. Several studies have analyzed the effect of VR in patients with neurological disorders, concluding that repetitive exercises and multisensory stimulation focused on DLAs stimulate motor evoked potentials, influencing corticospinal interactions in the premotor and motor areas of the brain [74]. This allows the reorganization of the neural networks that are responsible for controlling movement [75,76], due to increased activation of the primary motor cortex and the somatosensory cortex on the affected side [76,77]. Likewise, several studies in subjects who have suffered a stroke suggest that the use of non-immersive VR produces changes in the volume of gray matter in the motor and premotor regions of the affected extremity, which are correlated with the improvement of motor skills in brain areas that are not damaged. Specifically, correlations between changes in gray matter volume, increased range of motion of the extremity, and increased grip strength were found [78]. In addition, the use of VR and conventional therapy seems to generate a reduction in disabling motor symptoms such as spasticity [79], which can be explained by greater inhibitory control and a decrease in alpha neuron reflexes, which produces better motor control [80].

Several authors have affirmed that, when a VR device is adapted to the patient, benefits are obtained in terms of UE mobility [80] and strength [81]. In addition, it seems that the use of VR in children increases their motivation during therapy and favors the practice of physical activity within safe environments. This translates into greater adherence to treatment compared to other types of therapies [33]. Finally, this type of therapy can be applied at home, favoring the continuity of treatment over time [82]. This study presents some strengths. To begin, it is the first meta-analysis to assess the specific use of NWT to improve different motor and functional UE abilities in children with CP. Next, authors carried out a detailed synthesis of the tests employed in the studies included, allowing us to identify in each in each outcome whether NWT is effective. Finally, our meta-analysis points to NWT as a useful non-immersive VR device to be used in the UE recovery of children with CP. However, we cannot argue that NWT is better than other non-immersive VR devices. These findings may allow clinicians to make decisions about the employment of NWT to improve UE motor function in these patients.

This systematic review has several limitations. First, generalization of our results may be affected by the small number of RCTs included in each meta-analysis. Additionally, the small number of comparisons regarding the type of intervention in each meta-analysis markedly reduced the quality of the evidence in our findings. Likewise, the effect of therapy could only be assessed on the affected side due to the absence of data for the non-affected side. Another limitation is the small number of participants per study, which reduces the precision of the findings, despite the fact that the sample size tends to be low in this type of study. The inability to blind participants and therapists, and in some cases assessors, may have led to the presence of selection and classification bias in the studies included in the meta-analysis. In addition, variations in the trim-and-fill method of more than 10% indicate the presence of publication bias, which also makes it difficult to generalize our results. The low level of evidence in each meta-analysis is an important limitation that may affect the generalization of our findings. However, a strength of this study is that there are not enough studies published that clarify or replicate other researchers’ results, so the meticulous analysis of each measurement instrument used by each of the RCTs included in this review, as well as the identification of the domains and subscales of each of the instruments, allowed us to delimit the different outcomes to be analyzed within the greater construct that is UE function. This effort to analyze and identify outcomes makes this the first systematic review with meta-analysis to focus on UE functioning in this type of patient. It would be interesting in future research to carry out RCTs of high methodological quality, with a larger sample size, in order to analyze in depth this type of intervention in children with CP.

## 5. Conclusions

Our results showed that NWT can be considered effective for the treatment of UE motor function in children with CP. We also found that NWT plus conventional therapy may be better than conventional therapy to recover grip strength, tip grip strength, grasping ability, and UE-dissociated movements in children with CP. NWT is better than conventional therapy to improve tip grip strength and functional capacity in daily living activities and self-care in children with CP. Finally, NWT is better than no intervention to improve fine manual dexterity in children with CP, but no effectiveness of NWT alone or combined with conventional therapy was found for gross manual dexterity. Although more studies are needed to evaluate the effect of NWT on all outcomes related to UE function in children with CP, the findings presented in this meta-analysis may help clinicians to include NWT in the UE recovery of children with CP.

## Figures and Tables

**Table 1:** Bibliographical searches in each database.

Databases	Search Strategy
PubMed MEDLINE	(cerebral palsy[mh] OR cerebral palsy[tiab]) AND (virtual reality[mh] OR virtual reality[tiab] OR nintendo wii[tiab] OR wii[tiab] OR wii fit[tiab] OR wii balance board[tiab]) AND (upper extremity[mh] OR arm[mh] OR hand[mh] OR elbow[mh] OR wrist[mh] OR shoulder[mh] OR upper extremity[tiab] OR upper limb[tiab] OR arm[tiab] OR hand[tiab] OR elbow[tiab] OR shoulder[tiab] OR wrist[tiab])
Scopus	(TITLE-ABS-KEY (“cerebral palsy”) AND TITLE-ABS-KEY (“nintendo wii” OR “wii” OR “wii fit” OR “wii balance board” OR “virtual reality”) AND TITLE-ABS-KEY (“upper extremity” OR “upper limb” OR “arm” OR “hand” OR “elbow” OR “shoulder” OR “wrist”))
Web of Science	TOPIC:((cerebral palsy)) AND TOPIC:((nintendo wii OR wii OR wii fit OR wii balance board OR virtual reality)) AND TOPIC:((upper limb OR upper extremity OR arm OR hand OR elbow OR shoulder OR wrist))
PEDro	cerebral palsy, virtual reality
CINHAL Complete	(AB cerebral palsy) AND (AB virtual reality OR nintendo wii OR wii OR wii fit OR wii balance board) AND (AB upper extremity OR upper limb OR arm OR hand OR elbow OR shoulder OR wrist)

**Table 2:** Synthesis of the assessed outcomes and their measurements in studies included in the review.

Outcome	Definition	Measurements Used in each Selected Study
Grip strength (GS)	GS and tip GS refer to the maximum concentric force possessed by the hand muscles, as well as the force necessary to grasp small objects with the thumb and index finger, respectively	Manual or hydraulic dynamometer, measured in kilograms (kg) or Newton
Tip grip strength		
Functional capacity in daily living activities (dla) and self-care	Ability of an individual to perform DLAs and self-care tasks without the need for supervision, direction or assistance from other people	ABILHAND-kids
		“Self-care” domain of Pediatric Evaluation of Disability Index (PEDI)
		Children Health Assessment Questionnaire (CHAQ)
Upper extremity (ue) dissociated movements	Ability to move one part of the body (in this case the UE) independently of others	“Dissociated movements” domain of the Quality of Upper Extremity Skills Test (QUEST)
Gross motor dexterity (gmd)	GMD involves the movement of large muscle groups where precision of movements is not as important as in fine motor dexterity	Jebsen-Taylor Hand Function Test (JTHFT)
		Minnesota Manual Dexterity Test (MMDT)
		Box and Block test
Fine motor dexterity (fmd)	FMD involves the movement of small muscles that require eye-hand coordination to carry out very precise tasks	Movement Assessment Battery for Children-2 (MABC-2)
		Nine Hole Peg Test
		“Eye-hand coordination” of the Korean Developmental Test of Visual Perception (KDTVP)
Grasping ability	Ability of the hand to effectively grasp objects and to maintain a stable grip when the arm is in motion and in the absence of external forces	“Grip” domain of the QUEST

**Table 3:** Characteristics of the studies included in the review.

					Experimental Group									Control Group			
					Sample Characteristics					Experimental Intervention Characteristics				Sample Characteristics			Type Control Intervention
Author and Year	Country	Design	K	N	N_e_	Age (Range or Mean ± SD)	% Fem	Type of CP	GMFCS Level	Type	Dura- tion (weeks)	Session/Week	Minutes/Session	N_c_	Age (Range or Mean ± SD)	% Fem	
Acar, G et al. 2016 [57]	Turkey	RCT	1	30	15	9.53 ± 3.04	46%	Spastic hemiparesis	I/II	NWT + CT	6	2	15	15	9.73 ± 2.86	60	CT
Alsaif, A et al. 2015 [58]	Saudi Arabia	RCT	4	40	20	6–10 y	NR	Spastic bilateral hemiparesis	III	NWT	12	7	20	20	6–10 y	NR	NI
Atasavun-Uysal, SA et al. 2016 [59]	Turkey	RCT	1	24	12	9.13 ± 2.57	33%	Spastic unilateral	I/II	NWT + CT	12	2	30	12	NR	83%	CT
Avcil, E et al. 2020 [60]	Turkey	RCT	4	30	15	10.93 ± 4.09	46%	NR	I/II/III/IV	NWT + LMC	8	3	60	15	11.07 ± 3.24	40	CT
Chiu, HC et al. 2014 [61]	Australia	RCT	3	57	30	9.4 ± 1.9	50%	Spastic hemiplejic	I/II/III/IV/IV	NWT + CT	12	3	40	27	9.5 ± 1.9	59%	CT
El-Shamy, SM et al. 2018 [62]	Saudi Arabia	RCT	2	40	20	9.5 ± 1.2	40%	Spastic hemiplejic	NR	NWT + CT	12	3	40	20	9.8 ± 1.2	30%	CT
Sajan, JE et al. 2017 [63]	India	RCT	3	18	9	10.6 ± 3.78	40%	Spastic bilateral	I/II/III/IV	NWT + CT	3	6	45	9	12.4 ± 4.93	50%	CT
Shin, JW et al. 2015 [64]	South Korea	RCT	1	17	8	106.8 ± 2.5 months	63%	Spastic bilateral	NR	NWT	8	2	45	9	110.8 ± 16.1 months	33%	CT
Wang, TN et al. 2021 [65]	China	RCT	1	18	9	102.67 ± 25.05 months	66%	Spastic hemiplejic	NR	NWT + CT	8	2	145	9	102.78 ± 25.84 months	44%	CT

Abbreviations: K, Number of comparisons; N, Number of participants in study; N_e_, Number of participants in experimental group; N_c_, Number of participants in control group; SD, Standard Deviation; Fem, Female; CP, Cerebral Palsy; GMFCS, Gross Motor Function Classification System; RCT, Randomized Controlled Trial; NR, Not reported; NWT, Nintendo^®^ Wii Therapy; LMC, Leap Motion Controller; NI, No intervention; CT, Conventional Therapy.

**Table 4:** Risk of bias in the studies included in the review.

Study	Random Sequence Generation	Concealment of Randomization Sequence	Blinding of Participants	Blinding of Outcomes Assessors	Incomplete Outcome Data	Selective Reporting	Anything Else, Ideally Pre-Specified
Acar, G et al. 2016 [57]	+	+	+	+	-	-	-
Alsaif, A et al. 2015 [58]	-	+	+	+	?	-	-
Atasavun-Uysal, SA et al. 2016 [59]	-	-	+	+	-	-	-
Avcil, E et al. 2020 [60]	-	+	-	+	-	-	-
Chiu, HC et al. 2014 [61]	-	+	+	-	-	-	-
El-Shamy, SM et al. 2018 [62]	-	-	+	-	-	-	-
Sajan, JE et al. 2017 [63]	-	-	+	-	-	-	-
Shin, JW et al. 2015 [64]	-	+	+	+	?	-	-
Wang, TN et al. 2021 [65]	-	-	+	-	-	-	-

Abbreviations: “+” = high risk of bias, “-” = low risk of bias, “?” = inadequate data for the evaluation.

**Table 5:** Main findings in meta-analyses.

		Summary of Findings									Quality of Evidence (GRADE)					
		Pooled Effect						Publication Bias								
		K	N	N_s_	SMD	95% CI	I^2^	Funnel plot (Egger Test *p*-Value)	Trim and Fill		Risk of Bias	Incons	Indirect	Imprec	Pub. Bias	Quality
									Adj SMD	% of Change						
GRIP STRENGTH																
Specific NWT subgroups	NWT vs. CT	2	30	35	0.16	−0.56; 0.88	28.8%	NP	NP	NP	High	Possible	No	Yes	Possible	Very Low
	NWT + CT vs. CT	1	97	48.5	0.5	0.08; 0.91	52%	NP	NP	NP	Medium	Yes	No	Yes	Possible	Very Low
TIP GRIP STRENGTH																
Specific NWT subgroups	NWT vs. CT	1	30	30	0.8	0.01; 1.49	0%	NP	NP	NP	Low	No	No	Yes	Possible	Very Low
	NWT + CT vs. CT	1	40	40	0.95	0.3; 1.61	0%	NP	NP	NP	Medium	No	No	Yes	Possible	Very Low
FUNCTIONAL CAPACITY IN DLA AND SELF-CARE																
Specific NWT subgroups	NWT vs. CT	1	30	30	0.82	0.07;1.56	0%	NP	NP	NP	Medium	No	No	Yes	Possible	Very Low
	NWT + CT vs. CT	3	72	24	0.43	−0.04; 0.91	26%	0.66	0.43	0%	High	Possible	No	Yes	No	Low
UE DISSOCIATED MOVEMENTS																
Specific NWT subgroups	NWT + CT vs. CT	2	48	24	0.73	0.16; 1.3	0%	NP	NP	NP	High	No	No	Yes	Possible	Very Low
GROSS MANUAL DEXTERITY																
Specific NWT subgroups	NWT vs. CT	1	30	30	−0.12	−0.84; 0.6	0%	NP	NP	NP	Medium	No	No	Yes	Possible	Very Low
	NWT + CT vs. CT	3	105	35	−0.28	−0.67; 0.1	0%	0.88	−0.28	0%	High	No	No	Yes	No	Low
FINE MANUAL DEXTERITY																
Specific NWT subgroups	NWT vs. NI	1	40	40	3.12	1.53; 4.7	0%	NP	NP	NP	High	No	No	Yes	Possible	Very Low
	NWT + CT vs. CT	2	68	34	−0.05	−0.51; 0.41	0%	NP	NP	NP	Medium	No	No	Yes	Possible	Very Low
GRASPING ABILITY																
Specific NWT subgroups	NWT + CT vs. CT	2	48	24	0.72	0.14; 1.3	0%	NP	NP	NP	High	No	No	Yes	Possible	Very Low

Abbreviations: GRADE, Grading of Recommendations Assessment Development and Evaluation; Het, Heterogeneity; K, Number of studies; N, Number of participants in each meta-analysis; N_s_, mean of participants per study; SMD, Cohen Standardized Mean Difference; CI, Confidence Interval; I^2^, Higgins Degree of inconsistency; Adj, Adjusted; Incons, Inconsistency; Indirect, Indirectness; Imprec, Imprecision; Pub. Bias, Publication bias; Sym, Symmetric; Asym, Asymmetric; NWT, Nintendo^®^ Wii Therapy; CT, Conventional Therapy; DLA, Daily Living Activities; UE, Upper Extremity; NI, No Intervention; NP, Not possible to calculate.

## Data Availability

Not applicable.
